# Combined Treatment of Nitrated [6,6,6]Tricycles Derivative (SK2)/Ultraviolet C Highly Inhibits Proliferation in Oral Cancer Cells In Vitro

**DOI:** 10.3390/biomedicines10051196

**Published:** 2022-05-22

**Authors:** Sheng-Chieh Wang, Ching-Yu Yen, Jun-Ping Shiau, Meng-Yang Chang, Ming-Feng Hou, Jen-Yang Tang, Hsueh-Wei Chang

**Affiliations:** 1 Department of Biomedical Science and Environmental Biology, Ph.D. Program in Life Sciences, College of Life Sciences, Kaohsiung Medical University, Kaohsiung 80708, Taiwan; u107851101@gap.kmu.edu.tw (S.-C.W.); mifeho@kmu.edu.tw (M.-F.H.); 2 Department of Oral and Maxillofacial Surgery Chi-Mei Medical Center, Tainan 71004, Taiwan; ycy@tmu.edu.tw; 3 School of Dentistry, Taipei Medical University, Taipei 11031, Taiwan; 4 Department of Surgery, Kaohsiung Municipal Siaogang Hospital, Kaohsiung 81267, Taiwan; drshiaoclinic@gmail.com; 5 Division of Breast Oncology and Surgery, Department of Surgery, Kaohsiung Medical University Hospital, Kaohsiung Medical University, Kaohsiung 80708, Taiwan; 6 Department of Medicinal and Applied Chemistry, Kaohsiung Medical University, Kaohsiung 80708, Taiwan; mychang@kmu.edu.tw; 7 School of Post-Baccalaureate Medicine, Kaohsiung Medical University, Kaohsiung 80708, Taiwan; 8 Department of Radiation Oncology, Kaohsiung Medical University Hospital, Kaoshiung Medical University, Kaohsiung 80708, Taiwan; 9 Center for Cancer Research, Kaohsiung Medical University, Kaohsiung 80708, Taiwan

**Keywords:** dioxabicyclo[3.3.1]nonane core, ultraviolet C, oral cancer, synergy, apoptosis, DNA damage

## Abstract

Combined treatment is an effective strategy to improve anticancer therapy, but severe side effects frequently limit this application. Drugs inhibiting the proliferation of cancer cells, but not normal cells, display preferential antiproliferation to cancer cells. It shows the benefits of avoiding side effects and enhancing antiproliferation for combined treatment. Nitrated [6,6,6]tricycles derivative (SK2), a novel chemical exhibiting benzo-fused dioxabicyclo[3.3.1]nonane core with an *n*-butyloxy substituent, exhibiting preferential antiproliferation, was chosen to evaluate its potential antioral cancer effect in vitro by combining it with ultraviolet C (UVC) irradiation. Combination treatment (UVC/SK2) caused lower viability in oral cancer cells (Ca9-22 and OC-2) than single treatment (20 J/m^2^ UVC or 10 μg/mL SK2), i.e., 42.3%/41.1% vs. 81.6%/69.2%, and 89.5%/79.6%, respectively. In contrast, it showed a minor effect on cell viability of normal oral cells (HGF-1), ranging from 82.2 to 90.6%. Moreover, UVC/SK2 caused higher oxidative stress in oral cancer cells than normal cells through the examination of reactive oxygen species, mitochondrial superoxide, and mitochondrial membrane potential. UVC/SK2 also caused subG1 increment associated with apoptosis detections by assessing annexin V; panaspase; and caspases 3, 8, and 9. The antiproliferation and oxidative stress were reverted by *N*-acetylcysteine, validating the involvement of oxidative stress in antioral cancer cells. UVC/SK2 also caused DNA damage by detecting γH2AX and 8-hydroxy-2′-deoxyguanosine in oral cancer cells. In conclusion, SK2 is an effective enhancer for improving the UVC-caused antiproliferation against oral cancer cells in vitro. UVC/SK2 demonstrated a preferential and synergistic antiproliferation ability towards oral cancer cells with little adverse effects on normal cells.

## 1. Introduction

Oral cancer exhibits high morbidity and mortality globally [1,2,3]. The death rate in both genders increases year by year [4]. One primary reason for the high death rate in oral cancer cells is the late diagnosis. Except for surgery, chemotherapy and/or radiotherapy are alternative oral cancer treatments. However, chemotherapy and radiotherapy are often associated with adverse effects.

Cancer cells exhibit diverse responses and defenses to anticancer treatment. Multiple targeting strategies can improve the anticancer effects by using combined treatment. Natural products and clinical drugs were combined with radiation to enhance the antiproliferation effects on cancer cells [5,6,7]. Like chemotherapy and radiotherapy, the potential problems for combined treatment are the side effects that exhibit cytotoxicity to normal tissues and cells. To find selective antiproliferation in suitable combined treatment is necessary to improve the therapeutic effects against cancer.

Radiotherapy mainly focuses on ionizing radiation such as X-rays. However, the potential anticancer application of non-ionizing radiation such as ultraviolet C (UVC) shows antiproliferation effects on several cancers such as colon [8], breast [9], and oral [10] cancer cells. Moreover, mounting evidence has demonstrated that anticancer or chemical agents enhanced the UVC-induced antiproliferation against cancer cells, such as UVC/cisplatin to colon cancer cells [8], UVC/sulfonyl chromen-4-ones to oral cancer cells [10], and UVC/TiO_2_ microbead to urothelial cancer cells [11].

Dioxabicyclo[3.3.1]nonane core is a typical structure in many natural products [12,13,14,15,16]. The potential anticancer effects of dioxabicyclo[3.3.1]nonane core-containing chemicals have rarely been investigated. We recently developed a chemical exhibiting benzo-fused dioxabicyclo[3.3.1]nonane core with *n*-butyloxy substituent, namely, SK2, and first reported the antiproliferation effect on oral cancer cells [17]. However, the potential for enhancing UVC-induced antiproliferation of SK2 was not investigated.

The present study aims to evaluate the effects of UVC/SK2 combined treatment in the antiproliferation of oral cancer cells. Moreover, the normal cell response to UVC/SK2 was also considered. Mechanistically, oxidative stress, apoptosis, and DNA damage in modulating the responses of UVC/SK2 acting on oral cancer cells were explored.

## 2. Materials and Methods

### 2.1. SK2 Synthesis, Reagents, and UVC Irradiation

SK2 is a nitrated [6.6.6]tricycle with an *n*-butoxy substituted group. The detailed synthesis steps were previously mentioned [18]. Its shows >95% purity when examined by NMR analysis. SK2 was used before dissolving in dimethyl sulfoxide (DMSO), while *N*-acetylcysteine (NAC) (Sigma-Aldrich, St. Louis, MO, USA) [19,20,21] was dissolved in 1 X PBS. The medium was aspirated before UVC irradiation at lamina flow, which was operated by a germicidal lamp UVC (20 J/m^2^) for 20 s at a rate of 1 J/m^2^/s. The control was conducted the same way, but no UVC irradiation was performed. Subsequently, SK2 was incubated for the indicated time, as shown in the figure legend.

### 2.2. Cell Culture and Cell Viability

Ca9-22 oral cancer and HGF-1 normal oral cell lines were derived from the HSRRB (Osaka, Japan) and OC-2 oral cancer cell line was obtained by Dr. Wan-Chi Tsai (Kaohsiung Medical University, Taiwan) [22]. All cell lines belong to the biosafety level 1. DMEM/F-12 (Dulbecco’s modified Eagle’s medium (DMEM)/F-12) containing penicillin/streptomycin and 10% fetal bovine serum (Gibco; Grand Island, NY, USA) was used for cell maintenance. Promega MTS kit was applied to assess the 24 h cell viability (Madison, WI, USA) and detected at 490 nm by a multiplate reader.

### 2.3. Reactive Oxygen Species (ROS), Mitochondrial Superoxide (MitoSOX), and Mitochondrial Membrane Potential (MMP)

In a 37 °C incubator for 30 min, ROS reacted with 100 nM 2′,7′-dichlorodihydrofluorescein diacetate (H_2_DCF-DA) (Sigma-Aldrich) and MitoSOX reacted with 50 nM MitoSOX™ Red (Thermo Fisher Scientific, Carlsbad, CA, USA) [17]. The cellular signals for ROS and MitoSOX were assessed by Accuri C6 flow cytometry (Becton-Dickinson; Mansfield, MA, USA). MMP is proportional to the staining intensity of MitoProbe^TM^ DiOC_2_ (3). Cells were mixed with 5 nM MitoProbe^TM^ DiOC_2_ (3) (Thermo Fisher Scientific, Carlsbad, CA, USA) at 37 °C for 30 min [17]. The signals for MMP were assessed by flow cytometry.

### 2.4. Cell Cycle

Cells were mixed with 1 μg/mL DNA dye 7-aminoactinomycin D (7AAD) (Biotium Inc., Hayward, CA, USA) at 37 °C for 30 min. The signals for cellular DNA content were assessed by flow cytometry [17].

### 2.5. Apoptosis

According to user manuals, annexin V/7AAD [17], pancaspase [10], and Caspase-Glo^®^ 3/7 kits were applied to detect apoptosis, purchased from Strong Biotech Corp (Taipei, Taiwan), Abcam (Cambridge, UK), and Promega (Madison, WI, USA). The signals for pancaspase activity and annexin V/7AAD intensity were assessed by flow cytometry. Additionally, caspase 3/7 activity was detected by a microplate luminometer (Berthold Technologies GmbH and Co., Bad Wildbad, Germany) [10].

### 2.6. DNA Damages

After fixation, a primary antibody for p-Histone H2A.X (Ser 139) (Santa Cruz Biotechnology, Santa Cruz, CA, USA) (1:500) and Alexa 488-secondary antibody were added to cell suspensions. Then, cells were mixed with 5 μg/mL 7AAD. Finally, the signals of γH2AX and 7AAD detections were assessed by flow cytometry. Additionally, oxidative DNA damage 8-OHdG was reacted with the antibody against 8-hydroxy-2′-deoxyguanosine (8-OHdG)-FITC (Santa Cruz Biotechnology, Santa Cruz, CA, USA) (1:10,000) [10]. Finally, the signal of the 8-OHdG-FITC was assessed by flow cytometry.

### 2.7. Statistics

The significance of multiple comparisons was assessed by ANOVA with Tukey’s HSD tests. Data were shown as means ± SD (n = 3 repeats). Different assays included 4 or 8 treatments of the same cell lines. Data labeling with non-overlapped letters indicates a significant difference, while data labeling with overlapped letters indicates a nonsignificant difference.

## 3. Results

### 3.1. UVC/SK2 Combination Treatment Versus Single Treatment on Anti-Proliferation

**Figure 1:**
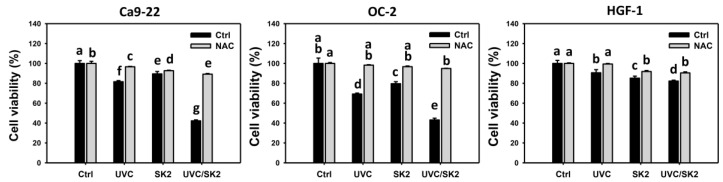
**Cell viability at 24 h MTS assay.** Oral cancer (Ca9-22 and OC-2) and normal oral (HGF-1) cells were treated with control, UVC (20 J/m^2^), SK2 (10 μg/mL), and UVC/SK2 (20 J/m^2^ and 10 μg/mL). Data indicate means ± SD (*n* = 4). The statistics of multiple comparisons were performed. The significance was judged from the connecting letters between different groups, i.e., showing a significant difference when the connecting letters were not overlapped (*p* < 0.05). For the example of OC-2 cells, the connecting letters for control, UVC, SK2, and UVC/SK2, showing ab, d, c, and e, indicate a significant difference between each other because they are not overlapped. Its untreated control and NAC control, showing ab and a, indicated a nonsignificant difference because they overlapped connecting letters.

Combination treatment (UVC/SK2) showed lower viability in oral cancer Ca9-22 cells than single treatment (20 J/m^2^ UVC or 10 μg/mL SK2), i.e., 42.3% vs. 81.6% and 89.5%, respectively (Figure 1). Similarly, UVC/SK2 showed lower viability in oral cancer OC-2 cells than single treatment (UVC or SK2), i.e., 43.1% vs. 69.2% and 79.6%. In contrast, it showed lower cytotoxicity in normal oral HGF-1 cells than in oral cancer cells. UVC/SK2 showed similar viability in oral cancer OC-2 cells compared to single treatment (UVC or SK2), i.e., 85.2% vs. 82.2% and 90.6%.

To assess the role of oxidative stress, the ROS inhibitor *N*-acetylcysteine (NAC) was pretreated before UVC/SK2 experiments. NAC recovered cell viability for these single or combined treatments to oral cancer and normal oral cells (Figure 1). The recovery effects of NAC in oral cancer cells (Ca9-22 and OC-2) dramatically changed back its viability to control. Therefore, UVC/SK2 can preferentially and synergistically inhibit oral cancer cells, showing minor damage to normal cells, depending on oxidative stress.

### 3.2. UVC/SK2 Combination Treatment Versus Single Treatment on ROS/MitoSOX

**Figure 2:**
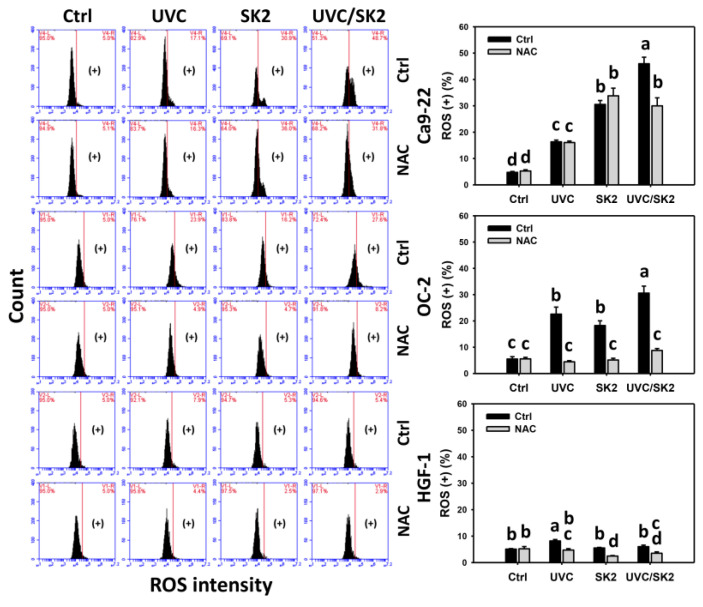
**ROS analysis.** Oral cancer (Ca9-22 and OC-2) and normal oral (HGF-1) cells were treated with control, UVC (20 J/m^2^), SK2 (10 μg/mL), and UVC/SK2 (20 J/m^2^ and 10 μg/mL). ROS (+) regions are marked with (+). Data indicate as means ± SD (*n* = 3). The statistics of multiple comparisons were performed. The significance was judged from the connecting letters between different groups, i.e., it showed a significant difference when the connecting letters were not overlapped (*p* < 0.05). For the example of Ca9-22 cells, the connecting letters for control, UVC, SK2, and UVC/SK2, showing d, c, b, and a, indicated a significant difference between each other because they were not overlapped. Its untreated control and NAC control, shown as d, indicated a nonsignificant difference because their connecting letters were overlapped.

**Figure 3:**
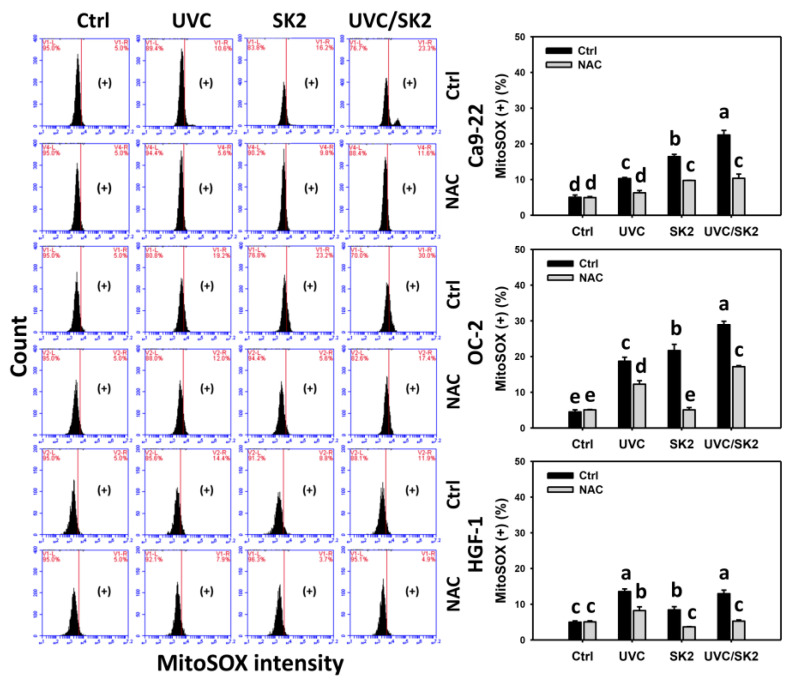
**MitoSOX analysis.** Oral cancer (Ca9-22 and OC-2) and normal oral (HGF-1) cells were treated with control, UVC (20 J/m^2^), SK2 (10 μg/mL), and UVC/SK2 (20 J/m^2^ and 10 μg/mL). MitoSOX (+) regions are marked with (+). Data indicate means ± SD (*n* = 3). The statistics of multiple comparisons were performed. The significance was judged from the connecting letters between different groups, i.e., it showed a significant difference when the connecting letters were not overlapped (*p* < 0.05). For the example of Ca9-22 cells, the connecting letters for control, UVC, SK2, and UVC/SK2, showing d, c, b, and a, indicated a significant difference between each other because they were not overlapped. Its untreated control and NAC control, showing d, indicated a nonsignificant difference because their connecting letters were overlapped.

The oxidative stress was examined by ROS and MitoSOX assays (Figure 2 and Figure 3, respectively). UVC/SK2 showed higher ROS levels in oral cancer cells (Ca9-22 and OC-2) than single treatment (Figure 2). In contrast, it showed lower ROS levels in normal oral HGF-1 cells than in oral cancer cells. Similarly, UVC/SK2 showed higher MitoSOX levels in oral cancer cells than a single treatment (Figure 3). In contrast, it showed lower MitoSOX levels in normal oral HGF-1 cells than in oral cancer cells.

Furthermore, NAC decreased ROS and MitoSOX levels of UVC/SK2 in oral cancer and normal oral cells (Figure 2 and Figure 3, respectively). Therefore, UVC/SK2 can preferentially and synergistically induce ROS and MitoSOX production in oral cancer cells, showing minor oxidative stress to normal cells, depending on oxidative stress.

### 3.3. UVC/SK2 Combination Treatment versus Single Treatment on MMP Destruction

**Figure 4:**
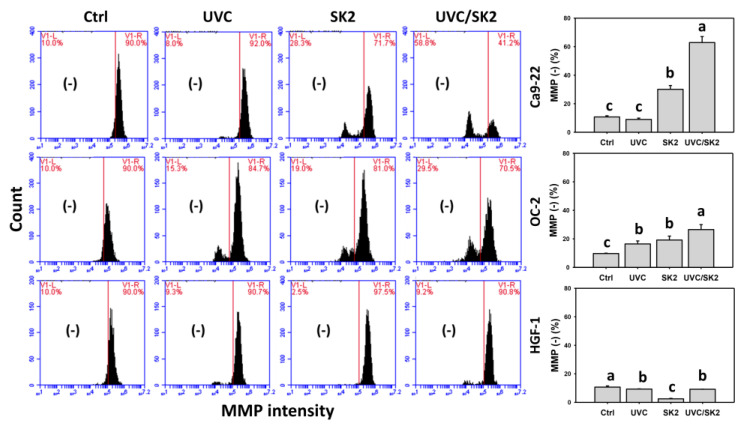
**MMP analysis.** Oral cancer (Ca9-22 and OC-2) and normal oral (HGF-1) cells were treated with control, UVC (20 J/m^2^), SK2 (10 μg/mL), and UVC/SK2 (20 J/m^2^ and 10 μg/mL). MMP (−) regions are marked with (−). Data indicate means ± SD (*n* = 3). The statistics of multiple comparisons were performed. The significance was judged from the connecting letters between different groups, i.e., it showed a significant difference when the connecting letters were not overlapped (*p* < 0.05). For the example of Ca9-22 cells, the connecting letters for UVC, SK2, and UVC/SK2, showing c, b, and a, indicated a significant difference between each other because they were not overlapped. Control and UVC, showing c, indicated a nonsignificant difference because their connecting letters were overlapped.

The oxidative stress was also examined by MMP assay (Figure 4). UVC/SK2 showed higher MMP (−) levels in oral cancer cells (Ca9-22 and OC-2) than in a single treatment (Figure 4). In contrast, it showed lower MMP (−) levels in normal oral HGF-1 cells than in oral cancer cells. Furthermore, NAC decreased MMP (−) levels of UVC/SK2 in oral cancer and normal oral cells (Figure 4). Therefore, UVC/SK2 can preferentially and synergistically induce MMP destruction in oral cancer cells, showing minor oxidative stress to normal cells, depending on oxidative stress.

### 3.4. UVC/SK2 Combination Treatment versus Single Treatment on subG1 Increment

**Figure 5:**
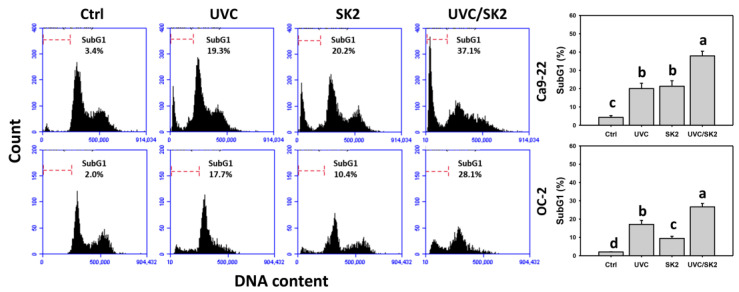
**Cell cycle distribution.** Oral cancer (Ca9-22 and OC-2) cells were treated with control, UVC (20 J/m^2^), SK2 (10 μg/mL), and UVC/SK2 (20 J/m^2^ and 10 μg/mL). Data indicate means ± SD (*n* = 3). The statistics of multiple comparisons were performed. The significance was judged from the connecting letters between different groups, i.e., it showed a significant difference when the connecting letters were not overlapped (*p* < 0.05). For the example of Ca9-22 cells, the connecting letters for control, UVC, and UVC/SK2, showing c, b, and a, indicated a significant difference between each other because they were not overlapped. UVC and SK2, showing b, indicated nonsignificant differences because their connecting letters were overlapped.

The apoptosis was primarily examined by cell cycle analysis (Figure 5). UVC/SK2 showed higher subG1 counts in oral cancer cells (Ca9-22 and OC-2) than in a single treatment (Figure 5). Therefore, UVC/SK2 can synergistically induce a subG1 Increment in oral cancer cells.

### 3.5. UVC/SK2 Combination Treatment versus Single Treatment on Apoptosis (Annexin V)

**Figure 6:**
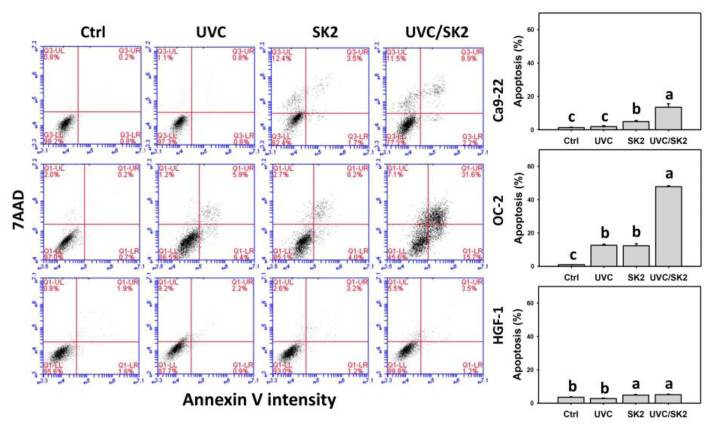
**Annexin V/7AAD assays.** Oral cancer (Ca9-22 and OC-2) and normal oral (HGF-1) cells were treated with control, UVC (20 J/m^2^), SK2 (10 μg/mL), and UVC/SK2 (20 J/m^2^ and 10 μg/mL). Annexin V (+)/7AAD (−) and annexin V (+)/7AAD (+) regions were assigned to apoptosis. Data indicate means ± SD (*n* = 3). The statistics of multiple comparisons were performed. The significance was judged from the connecting letters between different groups, i.e., it showed a significant difference when the connecting letters were not overlapped (*p* < 0.05). For the example of Ca9-22 cells, the connecting letters for UVC, SK2, and UVC/SK2, showing c, b, and a, indicated a significant difference between each other because they were not overlapped. Control and UVC, showing c, indicated a nonsignificant difference because their connecting letters were overlapped.

Since subG1 counts were increased, the potential apoptosis induction was further examined by annexin V assay (Figure 6). UVC/SK2 showed higher annexin V (+) counts (apoptosis) in oral cancer cells (Ca9-22 and OC-2) than in a single treatment (Figure 6). In contrast, it showed lower annexin V (+) counts in normal oral HGF-1 cells than in oral cancer cells. Therefore, UVC/SK2 can preferentially and synergistically induce apoptosis in oral cancer cells.

### 3.6. UVC/SK2 Combination Treatment versus Single Treatment on Caspase Activation

**Figure 7:**
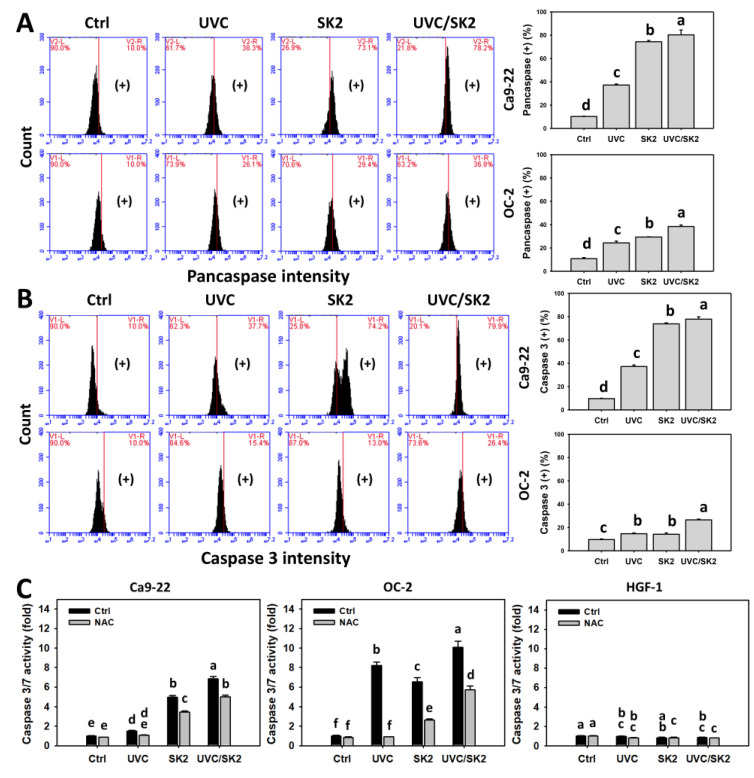
**Pancaspase, caspase 3, and caspase 3/7 assays.** Oral cancer (Ca9-22 and OC-2) and normal oral (HGF-1) cells were treated with control, UVC (20 J/m^2^), SK2 (10 μg/mL), and UVC/SK2 (20 J/m^2^ and 10 μg/mL). (**A**) Pancaspase and (**B**) caspase 3 (+) regions were marked with (+). (**C**) Caspase 3/7 activity. Data indicate means ± SD (*n* = 3). The statistics of multiple comparisons were performed. The significance was judged from the connecting letters between different groups, i.e., it showed a significant difference when the connecting letters were not overlapped (*p* < 0.05). For the example of (**A**) Ca9-22 cells, the connecting letters for control, UVC, SK2, and UVC/SK2, showing d, c, b, and a, indicated a significant difference between each other because they were not overlapped.

Since annexin V counts were increased, the potential apoptosis induction was further examined by detecting the caspase activation (Figure 7). The generic caspase assay (pancaspase) can detect the activation of most caspases. UVC/SK2 showed higher pancaspase counts in oral cancer cells (Ca9-22 and OC-2) than single treatment (Figure 7A).

The apoptosis executor caspase 3 was further examined by both flow cytometry (Figure 7B) and luminescence (Figure 7C) assays. UVC/SK2 showed higher caspase 3 counts in oral cancer cells than in single treatment (Figure 7B). Similarly, UVC/SK2 showed more increased caspase 3/7 activity in oral cancer cells than single treatment (Figure 7C). Moreover, UVC/SK2 showed higher caspase 3/7 activity in oral cancer cells than in normal oral cells. NAC downregulated these single and combined treatments for UVC/SK2-induced caspase 3/7 activation. Therefore, UVC/SK2 can preferentially and synergistically induce caspase activation in oral cancer cells, showing minor induction to normal cells, depending on oxidative stress.

### 3.7. UVC/SK2 Combination Treatment versus Single Treatment on Extrinsic and Intrinsic Signaling Activation

**Figure 8:**
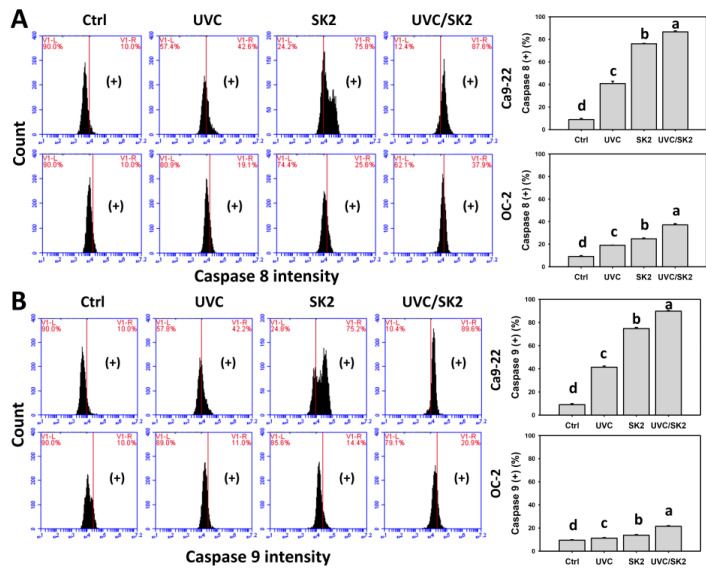
**Extrinsic and intrinsic apoptosis assays.** Oral cancer (Ca9-22 and OC-2) cells were treated with control, UVC (20 J/m^2^), SK2 (10 μg/mL), and UVC/SK2 (20 J/m^2^ and 10 μg/mL). (**A**) Caspase 8 (+) and (**B**) caspase 9 (+) regions were marked with (+). Data indicate means ± SD (*n* = 3). The statistics of multiple comparisons were performed. The significance was judged from the connecting letters between different groups, i.e., it showed a significant difference when the connecting letters were not overlapped (*p* < 0.05). For the example of (**A**) Ca9-22 cells, the connecting letters for control, UVC, SK2, and UVC/SK2, showing d, c, b, and a, indicated a significant difference between each other because they were not overlapped.

Caspase 8 and caspase 9, the extrinsic and intrinsic apoptotic caspases, were assessed by flow cytometry (Figure 8). UVC/SK2 showed higher caspase 8 and caspase 9 (+) counts in oral cancer cells (Ca9-22 and OC-2) than in single treatment (Figure 8A,B). Moreover, caspase 8 (+) counts were higher than caspase 9 (+).

### 3.8. UVC/SK2 Combination Treatment versus Single Treatment on DNA Damage

**Figure 9:**
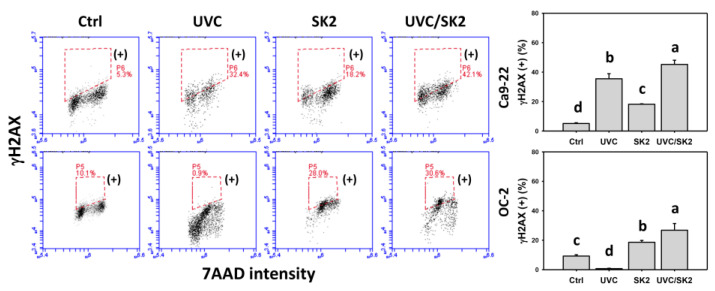
**γH2AXassays.** Oral cancer (Ca9-22 and OC-2) cells were treated with control, UVC (20 J/m^2^), SK2 (10 μg/mL), and UVC/SK2 (20 J/m^2^ and 10 μg/mL). Box regions are marked with (+). Data indicate means ± SD (*n* = 3). The statistics of multiple comparisons were performed. The significance was judged from the connecting letters between different groups, i.e., it showed a significant difference when the connecting letters were not overlapped (*p* < 0.05). For the example of Ca9-22 cells, the connecting letters for control, UVC, SK2, and UVC/SK2, showing d, b, c, and a, indicated a significant difference between each other because they were not overlapped.

**Figure 10:**
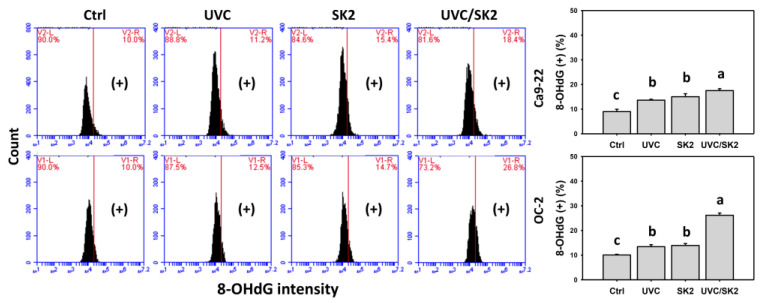
**8-OHdG assays.** Oral cancer (Ca9-22 and OC-2) cells were treated with control, UVC (20 J/m^2^), SK2 (10 μg/mL), and UVC/SK2 (20 J/m^2^ and 10 μg/mL). 8-OHdG (+) regions are marked with (+). Data indicate means ± SD (*n* = 3). The statistics of multiple comparisons were performed. The significance was judged from the connecting letters between different groups, i.e., it showed a significant difference when the connecting letters were not overlapped (*p* < 0.05). For the example of Ca9-22 cells, the connecting letters for control, SK2, and UVC/SK2, showing c, b, and a, indicated a significant difference between each other because they were not overlapped. UVC and SK2, showing b, indicated nonsignificant differences because their connecting letters were overlapped.

γH2AX and 8-OHdG were monitored by flow cytometry (Figure 9 and Figure 10, respectively). UVC/SK2 showed higher γH2AX and 8-OHdG (+) counts in oral cancer cells (Ca9-22 and OC-2) than in single treatment (Figure 9 and Figure 10).

## 4. Discussion

The potential combined treatment effects of dioxabicyclo[3.3.1]nonane core-containing chemicals to UVC have rarely been investigated. In the example of SK2, the antiproliferation performance of UVC/SK2 combined treatment was examined in the present study using oral cancer and normal oral cells. Several UVC/SK2-associated changes and mechanisms of oral cancer cells were discussed as follows.

The handicap for combined treatment is that some sensitizers may have toxicity to normal tissue, i.e., no preferential antiproliferation. Accordingly, it warrants the identification of drugs with a preferential antiproliferation to improve the synergistic effect of UVC treatment for cancer therapy. Several selective antiproliferation agents have been developed [23,24]. However, some selective antiproliferation agents have not been applied to the combined treatment. In the present study, we chose SK2 as the test chemical and found that UVC/SK2 shows a synergistic antiproliferation effect on oral cancer cells (Figure 1). Moreover, UVC/SK2 causes a selective antiproliferation effect on oral cancer cells but not on normal oral cells (Figure 1).

ROS is a well-known effector for the initiation of diverse cellular responses such as oxidative stress, apoptosis, DNA damage, endoplasmic reticulum stress, and autophagy [25,26], causing antiproliferation to cancer cells [27,28]. As expected, different treatments with oxidative-stress-generating functions may produce more oxidative stress in the combined application. In this view, combined treatment may synergistically inhibit proliferation through the synergistic generation of oxidative stress. UVC has the potential for oxidative stress [10,29] and apoptosis [30] in cancer cells. UVC also applies to anticancer using animal models [31,32,33]. Moreover, anticancer drugs often exhibit oxidative-stress-regulating function [27,28,34]. Accordingly, combined treatment with UVC and ROS-generating agents may cause synergistic antiproliferation to cancer cells.

SK2 is an oxidative-stress-generating agent showing ROS and MitoSOX induction in oral cancer cells [17]. UVC/SK2 combined treatment exhibited synergistic antiproliferation to oral cancer cells (Figure 1). Moreover, UVC/SK2 combined treatment induced higher oxidative stress (ROS, MitoSOX, and MMP) than independent treatment of oral cancer cells (Figure 2). Moreover, UVC/SK2-treated oral cancer cells exhibited higher ROS and MitoSOX than normal oral cells (Figure 2, Figure 3 and Figure 4). Accordingly, UVC/SK2 preferentially triggerred oxidative stress in oral cancer cells but not in normal oral cells. Additionally, the impacts of oxidative stress on the synergistic antiproliferation of UVC/SK2 in oral cancer cells were examined in the present study. NAC downregulated the UVC/SK2-promoted antiproliferation and ROS/MitoSOX burst in oral cancer cells (Figure 2 and Figure 3).

Oxidative stress is generally induced in ROS-generating natural products and clinical drugs [35]. This oxidative stress induction is partly attributed to downregulating antioxidant signaling [36]. For example, the nuclear-erythroid-like factor 2 (NRF2) was downregulated by fucoidan [37], and it, in turn, alleviated the cellular oxidative stress. Moreover, the downregulation of cellular antioxidants such glutathione may also contribute to the ROS generation in the example of fucoidan-treated oral cancer cells [37]. Accordingly, it warrants an in-depth assessment of the antioxidant regulation of UVC/SK2 in modulating synergistic oxidative stress and antiproliferation of oral cancer cells.

The ROS/MitoSOX-caused preferential burst of oxidative stress also triggered subG1 increment and apoptosis in oral cancer cells. This apoptosis induction was validated by pancaspase, caspase 3, and caspase 3/7 analysis (Figure 7). UVC/SK2 stimulated more caspase 8 (+) counts, which were higher than caspase 9 (+) in cancer cells (Ca9-22 and OC-2). These results suggest that UVC/SK2 preferentially triggers extrinsic apoptosis signaling compared to intrinsic apoptosis signaling in oral cancer cells. Moreover, UVC/SK2-treated oral cancer cells exhibited higher caspase 3/7 activity than normal oral cells (Figure 7C). Accordingly, UVC/SK2 preferentially triggers caspase activation in oral cancer cells but not in normal oral cells. Additionally, the impacts of oxidative stress on the synergistic caspase activation of UVC/SK2 in oral cancer cells were assessed in the present study. NAC downregulated the UVC/SK2-promoted caspase activation in oral cancer cells (Figure 7C).

In addition to ROS-generating drugs [38], oxidative stress also is stimulated in vitro [10,39,40,41] and in vivo [29] by radiations such as UVC. UVC also causes DNA damage such as γH2AX and 8-OHdG [10]. Combined UVC and ROS-generating drugs may increase the ROS level to improve the DNA damage and antiproliferation of cancer cells. SK2 is a ROS and 8-OHdG DNA damage-inducible chemical [17]. Similarly, UVC/SK2 causes higher γH2AX and 8-OHdG in oral cancer cells than individual treatments. Moreover, oxidative stress also suppresses the function of DNA repair [42,43]. Since the 8-OHdG is repaired by 8-oxoguanine glycosylase (OGG1) [43,44], it warrants a detailed assessment of DNA repair systems such as OGG1 of the UVC/SK2-treated oral cancer cells. In addition to DNA damage, UVC can attack macromolecules, leading to protein and lipid peroxidation [45]. Therefore, the impacts of UVC/SK2-generating oxidative stress on protein and lipid peroxidation cannot be excluded.

## 5. Conclusions

Nitrated [6,6,6]tricycles derivative (SK2) is a novel chemical exhibiting benzo-fused dioxabicyclo[3.3.1]nonane core with *n*-butyloxy substituent. Previously, we found the preferential antiproliferation and ROS-generating effects on oral cancer cells but not on normal oral cells [17]. However, the application for combined treatment of SK2 remains unclear, especially for connecting to UVC. In the present study, UVC/SK2 demonstrated the synergistic antiproliferation in oral cancer cells than individual treatments. UVC/SK2 also showed preferential antiproliferation to oral cancer cells but not normal oral cells. Moreover, UVC/SK2 exhibited synergistic inductions for oxidative stress, apoptosis, and DNA damage in oral cancer cells. Both UVC/SK2-promoted antiproliferation, oxidative stress, and apoptosis were rescued by NAC, indicating that the mechanism for synergistic antiproliferation plays a vital role in oxidative-stress-mediated responses of UVC/SK2 on oral cancer cells. Therefore, SK2 is a potential anticancer agent for improving the UVC-induced antiproliferation to oral cancer cells exhibiting minor cytotoxicity to normal oral cells.

## Data Availability

Data are contained within the article.

## Abbreviations

SK2	IUPAC name 6-n-butoxy-10-nitro-12,13-dioxa-11-azatricyclo[7.3.1.0^2,7^]trideca-2,4,6,10-tetrae
UVC	Ultraviolet C
ROS	Reactive oxygen species
MitoSOX	Mitochondrial superoxide
MMP	Mitochondrial membrane potential
NAC	*N*-Acetylcysteine
8-OHdG	8-Hydroxy-2′-deoxyguanosine
DMSO	Dimethyl sulfoxide
H_2_DCF-DA	2′,7′-Dichlorodihydrofluorescein diacetate
7AAD	7-Aminoactinomycin D
NRF2	Nuclear erythroid-like factor 2
